# Protection from oxygen–glucose deprivation by neurosteroid treatment in primary neurons and oligodendrocytes

**DOI:** 10.1007/s11626-024-00957-5

**Published:** 2024-07-29

**Authors:** Roisin Moloney, Carlton L. Pavy, Richard G. S. Kahl, Hannah K. Palliser, Jon J. Hirst, Julia C. Shaw

**Affiliations:** 1 School of Biomedical Sciences and Pharmacy, University of Newcastle, Newcastle, Australia; 2 Hunter Medical Research Institute, Mothers and Babies Research Centre, Newcastle, Australia

**Keywords:** Neurosteroid, Neuron, Oligodendrocyte, Preterm birth, GABA

## Abstract

Preterm birth results in an increased risk of neonatal brain injury and neurobehavioural disorders. Despite the seriousness of these adverse outcomes, there are currently no effective therapies to protect the vulnerable developing brain. We propose that neurosteroid replacement therapy may be a novel approach in reducing detrimental neurological outcomes following preterm birth. The use of guinea pig primary neuronal and oligodendrocyte cultures with relevance to late gestation allows insight into the mechanisms behind the effectiveness of these treatments. Primary neuronal and oligodendrocyte cultures were derived from fetal guinea pig frontal cortex brain tissue at gestational age 62 (GA62). Cell cultures were pre-treated with either etifoxine (5 µM) or zuranolone (1 µm) for 24 h prior to insult. Cells were then exposed to either oxygen–glucose deprivation (OGD; 0% O_2_ and no glucose DMEM; preterm birth insult) or sham (standard cell culture conditions; 25 mM DMEM) for 2 h. Lactate dehydrogenase assay (LDH) was performed following OGD as a measure of cytotoxicity. Relative mRNA expression of key neuronal and oligodendrocyte markers, as well as neuronal receptors and transporters, were quantified using high throughput (Fluidigm) RT-PCR. OGD significantly increased cellular cytotoxicity in both neurons and oligodendrocytes. Additionally, key neuronal marker mRNA expression was reduced following OGD, and oligodendrocytes displayed arrested mRNA expression of key markers of lineage progression. Treatment with etifoxine restored a number of parameters back to control levels, whereas treatment with zuranolone provided a robust improvement in all parameters examined. This study has demonstrated the neuroprotective potential of neurosteroid replacement therapy in a model of hypoxia related to preterm birth. Neuroprotection appears to be mediated through glutamate reduction and increased brain derived neurotrophic factor (BDNF). Future work is warranted in examining these treatments in vivo, with the overall aim to suppress preterm associated brain damage and reduce long term outcomes for affected offspring.

## Introduction

Preterm birth remains one of the most prevalent paediatric adversities, affecting an estimated 15 million infants every year (Blencowe *et al*. 2013). Whilst advances in healthcare have reduced mortality rates for these infants in resource rich nations such as Australia, preterm-born children are still at increased risk of adverse medical conditions, including hypertension, chronic kidney disease, cardiovascular disease, and neonatal infection (Samuel *et al*. 2019). Additionally, preterm born infants have a greater risk of neonatal brain injury, particularly due to hypoxic-ischemic (HI) events, which are associated with decreased oxygen and glucose supply. This occurs due to cerebral vascular fragility and immaturity of the preterm brain, as well as irregular blood pressure. Subsequently, preterm infants may experience ‘apnoea of prematurity’, which is defined as respiratory pauses associated with bradycardia, cyanosis and reduced oxygen saturation (Zhao *et al*. 2011).

Hypoxic-ischemic events trigger a cascade of downstream biochemical interactions that cause cellular dysfunction and eventually death. This occurs due to primary energy failure, related to hypoxia and impaired cerebral blood flow reducing oxygen and glucose levels, subsequently preventing oxidative phosphorylation from occurring (Allen and Brandon 2011). This in turn triggers disruption of the sodium–potassium pump (Na^+^/K^+^), leading to an excessive influx of sodium and a subsequent depolarization of neurons. This excessive depolarisation results in an increase in glutamate. Excitoxicity then results due to excessive glutamate levels and over-stimulation of excitatory receptors, leading to cell death (Khwaja and Volpe 2008).

In the second half of gestation, the brain is undergoing rapid changes that lead to marked vulnerability to excitotoxicity during this time. Brain volume more than doubles during this period, with cortical grey matter development increasing four-fold (Hüppi *et al*. 1998). Key developmental processes including the maturation of neuronal structures as well as the development of white matter and myelination pathways are also occurring. White matter development involves the proliferation and differentiation of oligodendrocytes, which are the cells responsible for myelin production. These cells mature along a developmental lineage, beginning with oligodendrocyte preprogenitors (OPPs), before maturing into oligodendrocyte precursor cells (OPCs). These are a proliferative pool of cells, that require factors such as platelet derived growth factor (PDGF) to continue to expand. Following withdrawal of these factors, OPCs differentiate into premyelinating oligodendrocytes (pre-OLs), and then immature OLs, which eventually become mature, myelinating oligodendrocytes (OLs) (van Tilborg *et al*. 2018). Cells of the earlier stages of oligodendrocyte development, such as OPCs and pre-OLs, are highly sensitive to damage particularly due to the increased iron content of the cells which renders them sensitive to excitotoxic damage (Butts *et al*. 2008). Preterm birth therefore dramatically increases the risk of white matter injury (WMI). This is proposed to result from an arrest in the maturation of the oligodendrocyte lineage, with post-mortem studies showing preterm-born infants with white matter injury were found to have an increased number of pre-OLs, but a reduction in immature oligodendrocytes and subsequent myelin (Buser *et al*. 2012).

Aberration in white matter development following preterm birth has been shown to involve the premature loss of neurosteroid levels (Shaw *et al*. 2021). Throughout gestation, the placenta produces progesterone, some of which is metabolised into the neurosteroid allopregnanolone. Neurosteroids are endogenous steroids that play critical roles in fetal neurodevelopment (Nguyen *et al*. 2003; Nicol *et al*. 1998). In late gestation, the fetus is maintained in what is known as the ‘fetal sleep state’, which is supported by the increased levels of the neurosteroid allopregnanolone. This allows for developmental processes such as myelination to occur appropriately, and also provides the developing brain with protection from excessive excitation and other damaging factors (Kelleher *et al*. 2011; Yawno *et al*. 2007). Allopregnanolone acts as an allosteric agonist of the GABA_A_ receptor, and through doing so increases inhibitory action in the fetal brain. Glial cells including oligodendrocytes also express GABA_A_ receptors and previous studies have indicated that allopregnanolone is able to stimulate the development of these cells (Faroni and Magnaghi 2011). Therefore, the premature loss of allopregnanolone may be a key mechanism behind the increased white matter injury and reduced maturation of oligodendrocytes in the preterm brain (Moloney *et al*. 2024a). Aberrations to oligodendrocytes and myelination have been associated with several neurobehavioural disorders such as ADHD, anxiety, and schizophrenia, of which the development of these disorders substantially increases following preterm birth (Breton *et al*. 2021; Onnink *et al*. 2015; Valdés-Tovar *et al*. 2022). Therefore, the development of neuroprotective treatments following preterm birth that can target oligodendrocytes and protect myelination are crucial to reduce brain damage and long term neurobehavioural disorders.

Current neuroprotective therapies for improving outcomes following preterm birth are limited. Clinical therapeutic hypothermia has been shown to improve outcomes for term born neonates (> 36 weeks gestation) with hypoxic-ischemic encephalopathy, if performed in tertiary Neonatal Intensive Care Unit (NICU) (Natarajan *et al*. 2018). In contrast the vulnerability and instability of preterm neonates results in a number of complications when administered clinical hypothermia that outweigh any benefits (Herrera *et al*. 2018). Other prospective treatments such as magnesium sulfate administration require antenatal administration, which prove limiting as more than 50% of preterm births are spontaneous (Menon 2008).

The current studies examine neurosteroid replacement therapy as a potential novel approach for neuroprotection of the preterm brain. Etifoxine is a non-benzodiazepine anxiolytic therapeutic currently used for the treatment of anxiety. This agent has a dual mechanism, through directly binding to the GABA_A_ receptor, and also indirectly modulating inhibitory action through stimulation of the translocator protein (TSPO). TSPO enables the movement of cholesterol into the mitochondrial matrix, thus facilitating the downstream synthesis of allopregnanolone (Lejri *et al*. 2019). Previous studies have demonstrated in an immortalised neuronal cell line model (PC-12 cell line) that etifoxine promoted the synthesis of protective glial-derived neurotrophic factor (Zhou *et al*. 2013). Zuranolone is another neurosteroid replacement therapy. Zuranolone is an allopregnanolone analogue that is currently in several clinical trials for major depression and anxiety and has recently been approved for post-partum depression (Barnes *et al*. 2023; Clayton *et al*. 2023; Deligiannidis *et al*. 2023; Kargbo 2023). Despite the promising results seen from these neurosteroid therapies, there is limited understanding of the mechanisms that may contribute to improving outcomes.

In this study we examine the neuroprotective effects of etifoxine and zuranolone in an in vitro model of preterm birth, using oxygen–glucose deprivation on primary neuronal and oligodendrocyte cells. We hypothesise that both etifoxine and zuranolone will exhibit neuroprotective abilities, that may warrant treatment with neurosteroid therapies to be a novel advancement in treatment following preterm birth.

## Methods

### Animals

All animal experiments and procedures in this study were conducted in accordance with the National Health and Scientific Research Council Australian Code of Practice for the Care and Use of Animals for Scientific Purposes and approved by the University of Newcastle Animal Care and Ethics Committee under ethics protocol A-2020–102. Tri-coloured outbred female guinea pig dams were obtained from the University of Newcastle Animal Services Unit. Dams were housed indoors under a 12-h light–dark cycle and were supplied with a diet of commercial guinea pig pellets, hay, and fresh vegetables. To ensure collection of the fetuses occurred at a specific time point, dams were post-partum time mated, and were euthanized via CO_2_ inhalation at gestational age 62 (GA62). For this study a total of 12 dams were used, with 6 male pups and 5 female pups collected.

### Isolation of primary neurons and oligodendrocytes-Collection of brain tissue

Isolation of primary neurons and oligodendrocytes from guinea pig tissue was performed as previously described (Moloney *et al*. 2024b). Briefly, post-partum time-mated guinea pig dams were culled as above, before an incision was made down the abdomen to expose the uterus. Pups were removed from the uterus and respective amniotic sacs before being heavily sprayed with 70% ethanol and decapitation performed. Heads were placed in ice cold PBS before transferring into a sterile cell culture hood. Heads were again heavily sprayed with 70% ethanol and placed in dissection medium on ice (see Table 1 for recipe) before removing the brain and isolating the frontal cortex. One frontal cortex section was minced into 1mm^3^ cubes, before separating half for neuronal isolation and half for oligodendrocyte isolation. One fetal brain was sufficient to produce four 12 well plates of each cell type.

**Table 1.** Reagents for cell culture

	Name	Company and Product Code
Media	Dulbecco’s modified Eagle’s media (DMEM) (high glucose, pyruvate, l-glutamine)	Gibco, Waltham, MA, 11965092
	Dulbecco’s modified Eagle’s media (DMEM) (no glucose, no pyruvate, l-glutamine)	Gibco 11966025
	Hank’s Balanced Salt Solution (HBSS)	Gibco 14175095
	Dulbecco’s Phosphate Buffered Solution (DPBS)	Gibco 14040133
	Neurobasal A Media	Gibco 10888022
Growth factors + additives	Papain	Worthington, Lakewood, NJ, LK003178
	Heat inactivated Horse serum	Merck, Darmstadt, Germany, H1138100ML
	Antibiotic–Antimycotic	Gibco 15240062
	Fetal bovine serum	Bovogen, Victoria, Australia SFBS-F
	HEPES	Gibco 15630080
	Glutamax	Gibco 35050061
	Sodium Pyruvate	Gibco 11360070
	Bovine serum albumin (BSA)	Sigma, Saint Louis, Missouri, a79061100G
	DNase	Sigma d5025375ku
	Recombinant human Fibroblast Growth Factor Basic (FGFb)	Gibco 2299059
	Human Apo-transferrin (APO-T)	Merck T1147500MG
	Insulin	Sigma SLCJ8080
	Recombinant Human Platelet-Derived Growth Factor (PDGF)	Australian BioSearch, Balcatta, Australia 773704
	Recombinant Human Epidermal Growth Factor (EGF)	Gibco 2209660
	Triiodothyronine (T3)	Merck T2877100MG
Plating reagents	Poly-L-ornithine	Sigma P0421100MG
	Poly-D-lysine	Sigma P08991G

### Isolation of primary neurons

Frontal cortex tissue was digested using papain (20U/ml) and DNase (0.2 mg/ml) in Hanks Balanced Salt Solution (HBSS) for 15 min in a rocking water bath. Following digestion, the cell pellet was washed in HBSS and then homogenized with a 10 ml serological pipette until the cell suspension was homogenous. The cell suspension was then passed through a 40 µM strainer and plated at 500 K/ml cells in poly-D-ornithine (50 µg/ml) coated 12 well tissue culture plates. Neurons were initially grown in a Neurobasal Plating Media for 24 h (refer to Table 1 for composition). To avoid overgrowth of astrocytes, the media was replaced with Neurobasal Feeding Media (Table 1) after 24 h. A 100% media change occurred every two days, and cells were collected at DIV7.

### Isolation of primary oligodendrocytes

As above, frontal cortex tissue was digested using papain (20U/ml) and DNase (0.2 mg/ml) in HBSS for 15 min in a rocking water bath. Following digestion, the cell pellet was then homogenized in DMEM20S (refer to Table 2 for recipe) with a 10 ml serological pipette until liquid was homogenous. Cells were passed through a 70 µm strainer and plated at 500 K/ml in poly-D-ornithine (50 µg/ml) coated T175 tissue culture flasks. The resulting cell population form oligodendrocyte progenitor cells (OPCs) upon a supportive astrocyte bed. Media was changed every 2 d until flasks reached 80–90% confluency. Following confluency, flasks were attached to a horizontal shaker at 200 rpm for 1 h to dislodge residual microglial cells. Media was then replaced, and flasks were reattached and shaken at 200 rpm for 18 h to allow for OPC’s to dislodge. The cell suspension was collected and placed in uncoated petri dishes in a standard cell culture incubator for 30 min to allow for any remaining microglia to adhere. The resulting cell suspension was then placed through a 40 µm strainer and plated at 80 K/ml in 12 well poly-D-ornithine coated plates (50 µg/ml) in OPC expansion media (refer to Table 1 for recipe). Cells received a 50% media change on DIV3, before a 100% media change on DIV6 to Maturation media (refer to Table 1 for recipe). Cells were collected on DIV13.

**Table 2.** Media recipes

Dissecting media	500 ml DMEM
	10 mM HEPES
	1% Antibiotic–antimycotic
Neurobasal plating media (NPM)	Neurobasal media
	2% B27 Supplement
	1% GlutaMax
	1% Antibiotic–antimycotic
	1 mM HEPES
	10% Heat Inactivated Horse Serum
Neurobasal feeding media (NFM)	Neurobasal media
	2% B27 Supplement
	1% GlutaMax
	1% Antibiotic antimycotic
	1 mM HEPES
DMEM20S	DMEM
	20% Fetal Bovine Serum
	1% antibiotic antimycotic
Oligodendrocyte Base Media	DMEM
	0.1% BSA
	50ug/mL Apo-transferrin
	5ug/mL insulin
	1% antibiotic antimycotic
	30 nM sodium selenite
	10 nM D-biotin
	10 nM hydrocortisone
OPC media	Oligodendrocyte Base Media
	20 ng/mL PDGF-AA
	20 ng/mL bFGF
	20 ng/ml EGF
Maturation media	Oligodendrocyte Base Media
	20ug/mL Triiodothyronine (T3)
Papain preparation	5 ml HBSS
	1 vial of papain (final concentration 20U/ml)

### Neurosteroid Treatment and Oxygen–Glucose Deprivation (OGD)

On DIV3 (neurons) or DIV6 (oligodendrocytes), cells were pre-treated with either Zuranolone (1 µM), Etifoxine (5 µM), or maturation media alone for 24 h prior to OGD. On DIV4 (neurons) or DIV7 (oligodendrocytes), OGD was performed by initially equilibrating glucose-free DMEM in the hypoxic chamber for 1 h to ensure oxygen was removed from the media. Cells were then placed into the air-tight chamber at 0% O_2_, 5% CO_2_ and 95% N_2_ for 2 h. Control cells received a media change of high-glucose DMEM, before returning to the standard cell incubator for the 2-h period. Following OGD, cells were removed from the hypoxic chamber and media was collected for LDH assessment. All cells then received their respective treatments in maturation media and were returned to the standard cell incubator.

### Lactate Dehydrogenase (LDH) Assay

Cellular cytotoxicity was assessed using the CyQUANT™ LDH Cytotoxicity Assay Kit (Invitrogen, Waltham, MA) by following the manufacturer’s instructions. Briefly, supernatant was collected from the neuronal (DIV4) and oligodendrocyte (DIV7) cells after OGD. 50ul of supernatant was transferred to a 96 well plate in triplicate, with absorbance measured at 490 nm and 680 nm using the Spectrostar Nano (BMG Labtech, Ortenberg, Germany). The maximum LDH able to be released from each cell type was determined per replicate by incubating a subset of cells in Lysis Buffer for 45 min prior to reading. Cytotoxicity values were determined by subtracting the 680 nm value (background signal) from the 490 nm value (true LDH) and normalizing to the maximum LDH value (with background subtracted).

### Real-Time PCR

RNA was extracted using the Bioline Isolate II Micro RNA kit (Meridian Bioscience; Cincinnati, OH), as per manufacturer’s instructions. Briefly, cells were collected into lysis buffer supplied with reducing agent tris (2-carboxyethyl) phosphine (TCEP). To assist with RNA recovery Carrier RNA (poly-A RNA: poly(A) potassium salt) was added at a final concentration of 20 ng/sample. Lysate was filtered to remove debris with DNase digestion performed on column. Total RNA was quantified with the Nanodrop™ One Spectrophotometer, with quality and purity of RNA determined using A260/A280 and A260/A230 ratios. Agarose gel electrophoresis was used as a further quality assessment. Synthesis of cDNA was performed using the Superscript IV Reverse Transcription kit (Invitrogen, Carlsbad, CA) with random hexamers using a GeneAmp 9700 PCR Machine.

Primer sequences were designed and optimised for the guinea pig by our laboratory and are detailed in Table 3. Gene data expression was obtained using the Fluidigm Juno and Biomark systems as previously described (Crombie *et al*. 2021, 2023). First, samples were preamplified using the PreAmp Master Mix according to manufacturer’s instructions. Primers were prepared (0.5 pmol/uL) in EVAGreen and RT-PCR was performed using the Biomark HD system. A calibrator of pooled brain tissue samples was used to ensure consistency between plates. Results were analysed using the comparative CT method of analysis normalised to the housekeeping genes *ACTB*, *TBP*, *YWHAZ*, *UBE2D2*.

**Table 3.** Guinea pig specific primers for real time PCR

Gene ID	Protein	Forward Primer	Reverse Primer	Amplicon Size (bp)
*DRD1*	Dopamine Receptor D1	ACCTCCAGCATGGATGAGAC	TGACAGGAAACAGGCTGTCA	78
*DRD2*	Dopamine Receptor D2	CCTGCCAAGCCAGAGAAGAA	GGGCATGGACTGGATCTCAAA	78
*GABRA1*	GABAA α1 Receptor	CTCAAGCCCGCAATGAAGAAA	TCCAGTCAACGTGCTCAGAA	81
*GABRA2*	GABAA α2 Receptor	ACTAGGCCAATCAATTGGGAA	TCAAGTGGAAATGAGCTGTCA	80
*GABRA3*	GABAA α3 Receptor	TTGGCAGCTATGCCTACACA	ACCTCCACAGACTTGTTCTTCC	73
*GABRA4*	GABAA α4 Receptor	TGG​ACA​AAG​GGT​CCT​GAG​AAA	CAC​TGT​TTG​CCC​AAT​CAG​ATC​A	84
*GABRA5*	GABAA α5 Receptor	TGGTTCATCGCTGTGTGCTA	CCCAGCCTCTCTTCGTGAAATA	85
*GABRAD*	GABAA Delta Receptor	ATG​CTG​GAC​CTG​GAA​AGC​TA	GGA​TCT​GCT​CCT​GGT​TCT​CA	76
*GAD67*	Glutamate Decarboxylase 1 (67 kDa)	AGC​TCG​CTA​CAA​GTA​CTT​CCC	TGT​GTT​CTG​AGG​TGA​AGA​GGA​C	83
*SLC1A2*	Excitatory amino acid transporter 3 (EAAT3)	CAC​AGT​CGT​CTC​CCT​GTT​GAA	CAG​GCC​CTT​CTT​GAG​AAC​CA	76
*RBFOX3*	RNA Binding Fox-1 Homolog 3 (NeuN)	CACAGACAGACAGCCAACCA	CGGAAGGGGATGTTGGAGAC	88
*PVALB*	Parvalbumin	AAGGATGGGGACGGCAAA	GGGTCCATCAGCTCTGCTTA	77
*CALB1*	Calbindin	CTGACTGAGATGGCCAGGTTA	CCCACACATTTTAACTCCCTGAAA	75
*SST*	Somatostatin	AAGCAGGAACTGGCCAAGTA	TGGGACAAATCTTCAGGTTCCA	92
*GRIA1*	Glutamate Ionotropic Receptor AMPA Type Subunit 1	TGAACGCAGGACTGTCAACA	AAGCTCGGTGTGATGAAGCA	72
*GRIA2*	Glutamate Ionotropic Receptor AMPA Type Subunit 2	GACACCTCACATCGACAACC	CGCCTCTTGAAAACTGGGAA	80
*GRIN1*	Glutamate Ionotropic Receptor NMDA Type Subunit 1	AGAGCATCCACTTGAGCTTCC	TACACGCGCATCATCTCGAA	82
*GRIN2A*	NMDA 2A Receptor	TCGAGGATGCGAAGACACAA	AGCCTCGTCTTTGGAGCAATA	80
*GRIN2D*	NMDA 2D Receptor	ACCTGGGATAACCGGGACTA	TGTCTCTGGTGAGGGAAATGAC	85
*GFAP*	Glial fibrillary acidic protein	AAGAGGCATCCAGCTACCAG	GGTAGGTGGCAATCTCGATGT	81
*OLIG2*	Oligodendrocyte transcription factor	GCACTCATCCTGGGGACAA	CCGACGACGTGGATGATGAA	78
*NCAM1*	Neural cell adhesion molecule 1	TTGTTCCCAGCCAAGGAGAA	TGTCTTTGGCATCTCCTGCTA	78
*CSPG4*	Chondroitin sulfate proteoglycan 4	CTCCTCACCACCACCCTCAA	ACTCTTCAGCACAGCCCTCA	79
*GALC*	Galactosylceramidase	ACTTCCCGCCTTCTGGTAAA	AGGTTCAGTGCCATCTGTTGT	144
*MBP*	Myelin Basic Protein	ACCTCCTCCGTCTCAAGGAAA	GCTCTGCCTCCATAGCCAAA	66
*BDNF*	Brain derived neurotrophic factor	AATCGGCTGGCGGTTCATAA	AGCCACTATCTGCCCCTCTTA	75
*ACTB*	Housekeeper	TGCGTTACACCCTTTCTTGAC A	ACAAAGCCATGCCAATCTCAT	72
*YHWHAZ*	Housekeeper	GCTTCACAAGCAGAGAGCAA	CAGCAACTTCGGCCAAGTAA	76
*TBP*	Housekeeper	CAAGCGGTTTGCTGCTGTAA	CACCATCTTCCCGGAACTGAA	79
*UBE2D2*	Housekeeper	CAGTGCTGCGTGTTGTACATA	TGCTAGGAGGCAATGTTGGTA	77

### Statistical Analysis

Data was analysed using Prism v9.0 (Graphpad Software Inc., La Jolla, CA) and is presented as mean ± SEM for each group with significance considered *p* < 0.05. Relative expression was determined by normalising each condition to its respective control cell value. For this study, all samples represent an individual biological replicate obtained from one animal. Cells were plated in triplicate per animal and were pooled for collection. Normality of the samples was assessed using Brown-Forsythe tests. Significance was assessed using Kruskal–Wallis one way ANOVA, with Dunn’s multiple comparisons used to assess changes between all groups.

## Results

### Zuranolone reduces OGD-induced cytotoxicity in neurons and oligodendrocytes

**Figure 1.**
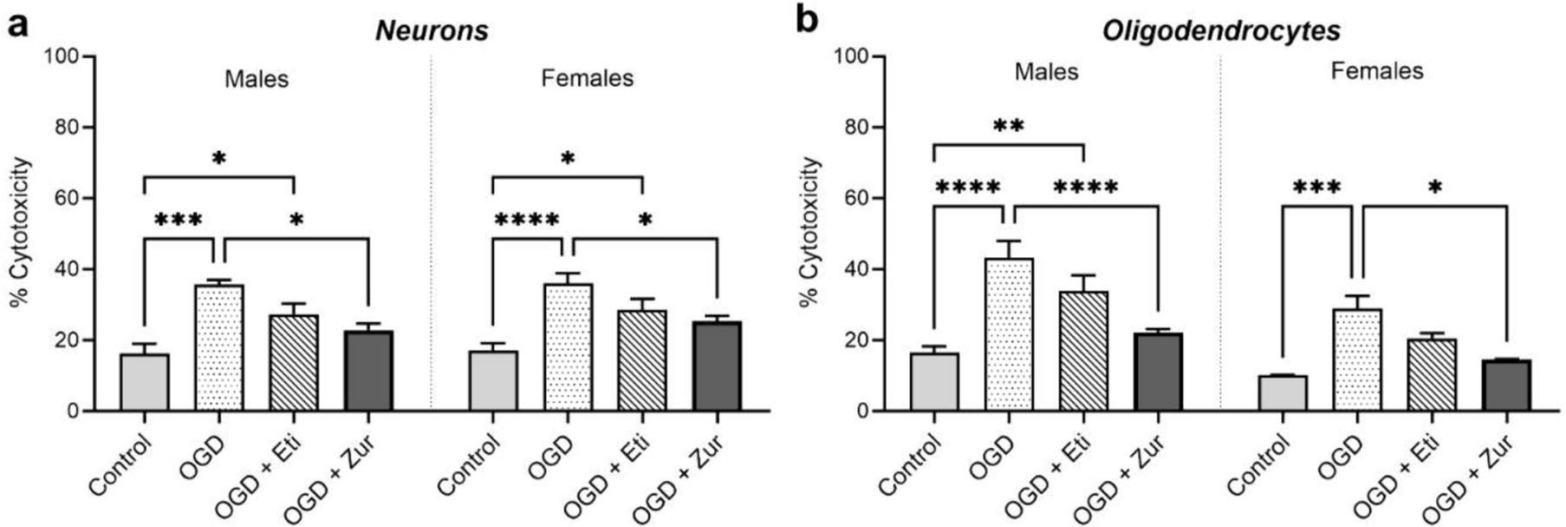
*Cytotoxicity % of primary neuronal and oligodendrocyte cultures after OGD insult*. Cytoxicity was quantified in (*a*) neurons and (*b*) oligodendrocytes following sham or OGD. Data presented as mean ± SEM with control (light grey; *n* = 5/sex), OGD (spotted; *n* = 5/sex), OGD treated with etifoxine (striped; *n* = 5/sex), and OGD treated with zuranolone (dark grey; *n* = 5/sex) groups and * indicating significance at *p* < 0.05, *** indicating significance at *p* < 0.001 and **** indicating *p* < 0.0001.

In primary neurons, OGD caused a significant increase in cytotoxicity in males and females (*p* = 0.0001, *p* < 0.0001; Fig. 1*a*). This increase was sustained in both sexes following etifoxine treatment (*p* = 0.04, *p* = 0.01). However, zuranolone treatment significantly reduced cytotoxicity in males (*p* = 0.01) and females (*p* = 0.02). Similar responses were observed in the oligodendrocyte culture, with OGD oligodendrocytes having a significant increase in cytotoxicity in both sexes (*p* < 0.0001, *p* = 0.0004; Fig. 1*b*), which was sustained in etifoxine treated male oligodendrocytes (*p* = 0.0012), but not females. Zuranolone treatment significantly reduced cytotoxicity in both sexes in oligodendrocyte cultures (*p* < 0.0001, *p* = 0.01).

### Effects of neurosteroid therapy on neurons exposed to OGD insult-mRNA expression of neurotrophic factor and GABAergic interneurons is reduced with OGD and restored with neurosteroid treatment

**Figure 2.**
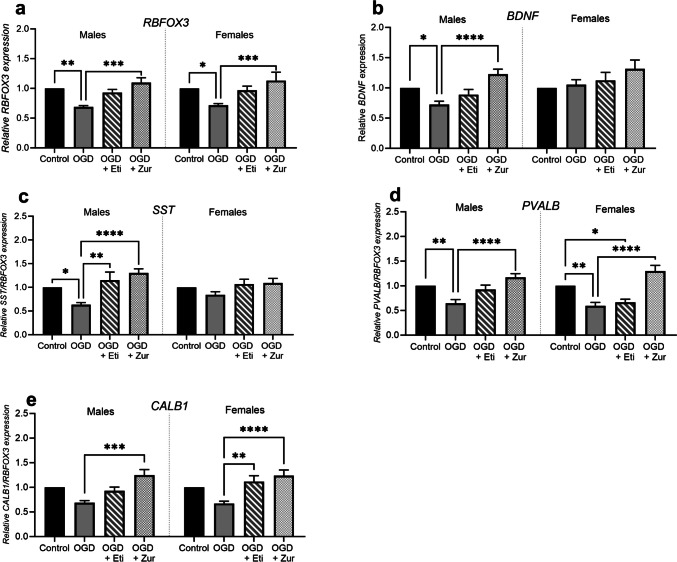
*mRNA expression of neuronal markers following OGD and neurosteroid therapy treatment in neurons*. Neurons are shown as control (black; *n* = 5/sex), OGD (dark grey; *n* = 5/sex), OGD treated with etifoxine (striped; *n* = 5/sex), OGD treated with zuranolone (light grey spotted; *n* = 5/sex). Neuronal mRNA expression includes *RBFOX3 *(*a*), *BDNF *(*b*), *SST *(*c*), *PVALB *(*d*) and *CALB1 *(*e*). Relative mRNA expression is normalized to respective control cells. Data is presented as mean ± SEM with * indicating significance at *p* < 0.05, ** indicating *p* < 0.01, *** indicating *p* < 0.001 and **** indicating *p* < 0.0001.

RNA binding protein fox-1 homolog 3* (RBFOX3)*, a marker of overall neuronal expression, was significantly reduced following OGD insult in both sexes compared to control (*p* = 0.008, *p* = 0.02; Fig. 2*a*). This was then increased following zuranolone treatment (*p* = 0.0003, *p* = 0.0004 for males and females respectively), but not with etifoxine.

Brain-derived neurotrophic factor (*BDNF*), a key promoter of neuronal survival and growth, was found to be significantly reduced in male neuronal cultures exposed to OGD insult when compared to control (*p* = 0.03, Fig. 2*b*). Once again, this was restored in male cells treated with zuranolone and exposed to OGD (*p* < 0.0001), but not etifoxine. In contrast, in female culture, no significant changes were observed.

The expression of GABAergic interneuron markers somatostatin (*SST*), parvalbumin (*PVALB*), and calbindin (*CALB1*) was also quantified. Importantly, their results were normalized to their respective *RBFOX3* values prior to normalizing to respective controls to ensure OGD insult related changes were due to the insult and not the overall global loss of neurons.

*SST* expression was significantly reduced in male neurons exposed to OGD when compared to control (*p* = 0.01, Fig. 2*c*). Interestingly, expression was significantly increased in both etifoxine and zuranolone treated male cells exposed to OGD compared to insult alone (*p* = 0.001, *p* < 0.0001). *PVALB* expression showed a significant reduction following OGD insult in both sexes (*p* = 0.006, *p* = 0.002; Fig. 2*d*). This reduction remained in etifoxine treated female neurons exposed to OGD (*p* = 0.02). Alternatively, zuranolone treated male and female neurons exposed to OGD insult were found to have a significant increase in *PVALB* expression compared to OGD insult alone (*p* < 0.0001, *p* < 0.0001). Lastly, *CALB1* expression appeared to decrease following OGD insult in both sexes, however, did not reach significance (*p* = 0.09, *p* = 0.06; Fig. 2*e*). There was a significant increase in expression of *CALB1* in male zuranolone treated neurons exposed to OGD (*p* = 0.0002), and an increase in both female etifoxine and zuranolone treated neurons exposed to OGD (*p* = 0.003, *p* < 0.0001, respectively).

### Neurosteroid therapies increase mRNA expression of specific GABA_A_receptor subunits

**Figure 3.**
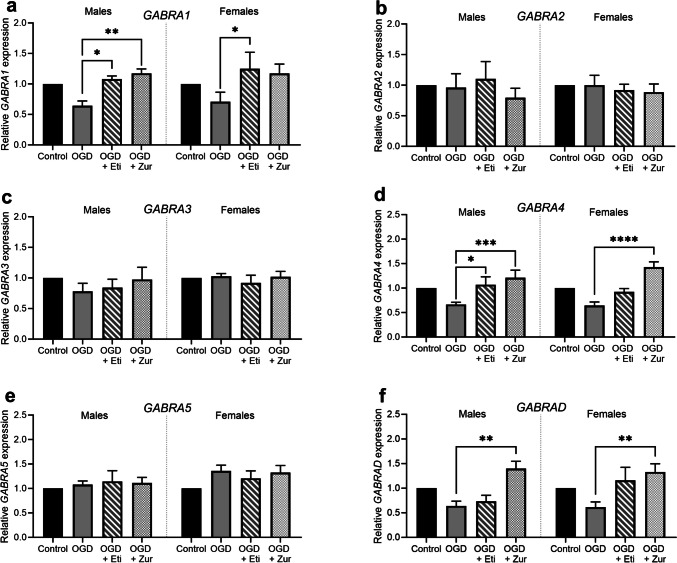
mRNA expression of GABA_A_ receptor subunits following OGD and neurosteroid therapy treatment in neurons. Neurons are shown as control (*black*; *n* = 5/sex), OGD (*dark grey*; *n* = 5/sex), OGD treated with etifoxine (*striped*; *n* = 5/sex), OGD treated with zuranolone (*light grey* spotted; *n* = 5/sex). GABA_A_ mRNA expression includes *GABRA1* (*a*), *GABRA2* (*b*), *GABRA3* (*c*), *GABRA4* (*d*), *GABRA5* (*e*) AND *GABRAD*. Relative mRNA expression is normalized to respective control cells. Data is presented as mean ± SEM with * indicating significance at *p* < 0.05, ** indicating *p* < 0.01, *** indicating *p* < 0.001 and **** indicating *p* < 0.0001.

Treatment with etifoxine and zuranolone significantly increased *GABRA1* expression in males compared to insult alone (*p* = 0.04, *p* = 0.006; Fig. 3*a*). Etifoxine, but not zuranolone (*p* = 0.06), treatment also increased *GABRA1* expression in females compared to OGD insult alone (*p* = 0.02). Similarly, *GABRA4* was also increased in etifoxine and zuranolone treated male cells (*p* = 0.01, *p* = 0.03; Fig. 3*d*), and in zuranolone treated female cells (*p* < 0.0001), compared to their OGD insult only counterparts. Finally, *GABRD* mRNA expression was also increased in zuranolone treated male and female neuronal cultures (*p* = 0.002, *p* = 0.005; Fig. 3*f*). There were no other significant differences identified (Fig. 3*b*, *c*, *e*).

### Effect of OGD and neurosteroid treatment on mRNA expression of glutamatergic receptors

**Figure 4.**
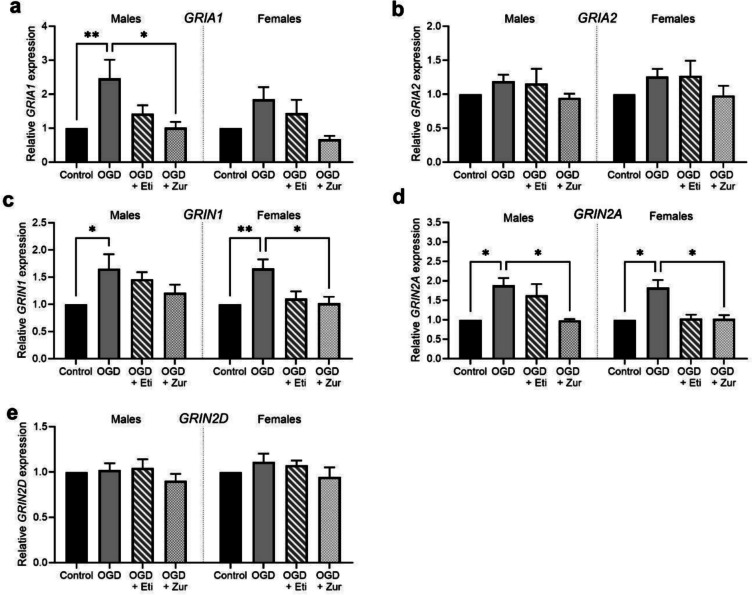
mRNA expression of glutamatergic receptor subunits following OGD and neurosteroid therapy treatment in neurons. Neurons are shown as control (*black*; *n* = 5/sex), OGD (*dark grey*; *n* = 5/sex), OGD treated with etifoxine (*striped*; *n* = 5/sex), OGD treated with zuranolone (*light grey* spotted; *n* = 5/sex). Glutamatergic receptor mRNA expression includes *GRIA1 *(*a*), *GRIA2 *(*b*), *GRIN1 *(*c*), *GRIN2A *(*d*) and *GRIN2D* (*e*). Relative mRNA expression is normalized to respective control cells. Data is presented as mean ± SEM with * indicating significance at *p* < 0.05 and * indicating *p* < 0.01.

AMPA receptor subunit 1 (*GRIA1)* was significantly increased in male OGD insulted cells compared to control (*p* = 0.005; Fig. 4*a*), with zuranolone treatment preventing this increase (*p* = 0.02). Treatment with etifoxine also appeared to reduce *GRIA1* expression in male cells however this did not reach significance (*p* = 0.1). There were no significant changes in *GRIA1* expression observed in female neurons, and no significant changes in either sex for *GRIA2* expression (Fig. 4*b*).

In both male and female OGD exposed cultures, mRNA expression of NMDA receptor subunit 1 (*GRIN1)* was significantly increased compared to control (*p* = 0.01, *p* = 0.009; Fig. 4*c*). In female neuronal cultures this was prevented by zuranolone treatment (*p* = 0.03) but not etifoxine (*p* = 0.08).

For both male and female cultures, OGD significantly increased expression of NMDA receptor subunit 2A (*GRIN2A*) compared to control (*p* = 0.002, *p* = 0.01; Fig. 4*d*). This was prevented by zuranolone treatment in males and females (*p* = 0.02, *p* = 0.03). Etifoxine treatment also appeared to prevent this increase in female cultures, but it did not reach significance (*p* = 0.07).

No significant differences were observed for NMDA receptor subunit 2D (*GRIN2D*) expression (Fig. 4*e*).

### Neurosteroid therapies restore dopamine receptor mRNA expression following OGD

**Figure 5.**
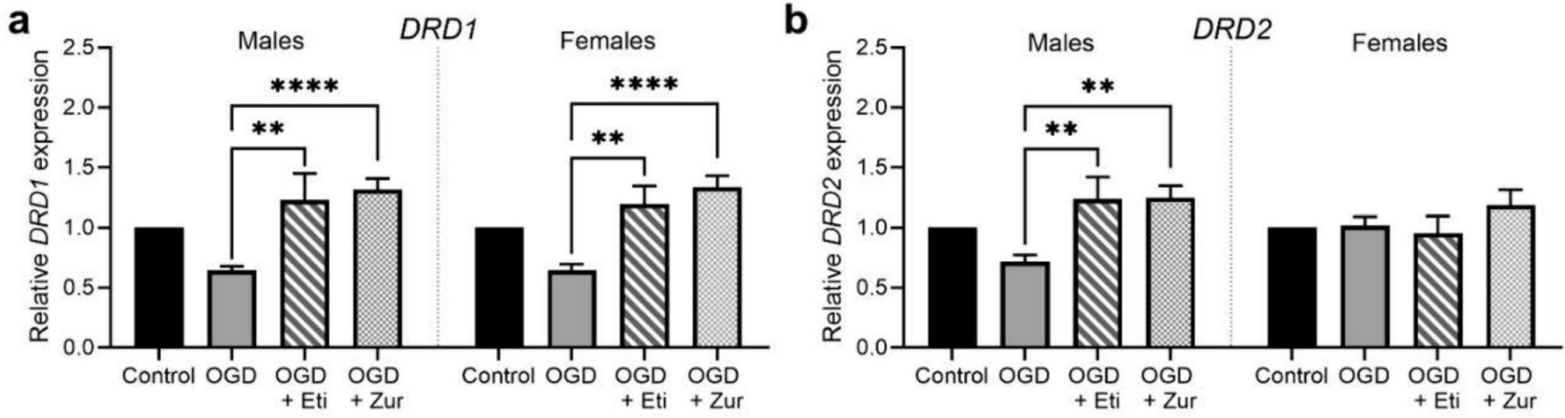
mRNA expression of dopamine receptor subunits following OGD and neurosteroid therapy treatment in neurons. Neurons are shown as control (*black*; *n* = 5/sex), OGD (*dark grey*; *n* = 5/sex), OGD treated with etifoxine (*striped*; *n* = 5/sex), OGD treated with zuranolone (*light grey* spotted; n = 5/sex). Dopamine receptor mRNA expression includes *DRD1 *(*a*) and *DRD2 *(*b*). Relative mRNA expression is normalized to respective control cells. Data is presented as mean ± SEM with * indicating significance at *p* < 0.05, * indicating significance at *p* < 0.01 and **** indicating < 0.0001.

Compared to OGD insult only cells, mRNA expression of dopamine receptor D1 (*DRD1*) was significantly increased with both etifoxine and zuranolone treatment in males (*p* = 0.0014, *p* < 0.0001) and females (*p* = 0.002, *p* < 0.0001; Fig. 5*a*). For dopamine receptor D2 (*DRD2*) expression, OGD exposed males had a significant increase in expression following etifoxine and zuranolone treatment compared to the OGD insult only group (*p* = 0.002, *p* = 0.001; Fig. 5*b*), but there were no significant differences observed in females.

### mRNA expression of GABA synthesis and glutamate transport is modulated by neurosteroid treatment

**Figure 6.**
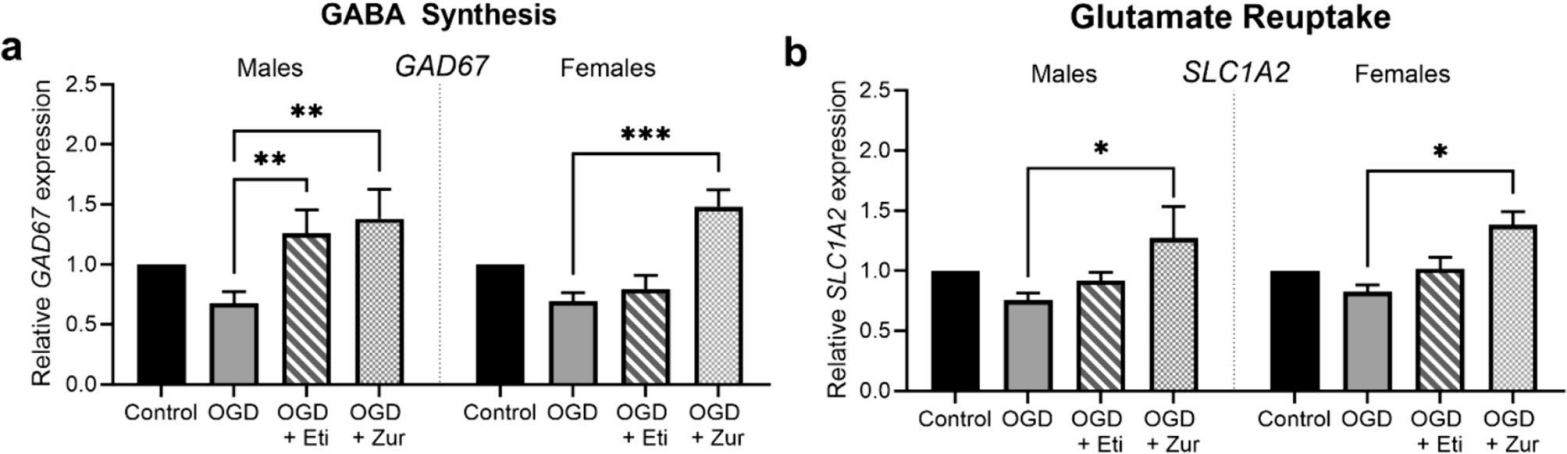
mRNA expression of GAD67 and SLC1A2 following OGD and neurosteroid therapy treatment in neurons. Neurons are shown as control (*black*; *n* = 5/sex), OGD (*dark grey*; *n* = 5/sex), OGD treated with etifoxine (*striped*; *n* = 5/sex), OGD treated with zuranolone (*light grey* spotted; *n* = 5/sex). Relative mRNA expression is normalized to respective control cells. Data is presented as mean ± SEM with * indicating significance at *p* < 0.05 and ** indicating *p* < 0.01.

Expression of glutamate decarboxylase (*GAD67*), the enzyme responsible for the synthesis of GABA from glutamate, was significantly increased in male cells treated with etifoxine and zuranolone (*p* = 0.001, *p* = 0.008 Fig. 6*a*), and in female cells treated with zuranolone (*p* = 0.0002), compared to insult only cells.

Similarly, expression of excitatory amino acid transporter EAAT2 (*SLC1A2*), one of the key glutamate transporters that removes glutamate from the synaptic cleft, was also significantly increased by zuranolone treatment in male and female cells exposed to OGD (*p* = 0.02, *p* = 0.04; Fig. 6*b*) compared to insult only.

### Effects of neurosteroid treatments on oligodendrocytes exposed to OGD-mRNA expression of stage specific oligodendrocyte markers is restored with neurosteroid treatments-mRNA expression of stage specific oligodendrocyte markers is restored with neurosteroid treatments

Neural cell adhesion molecule (*NCAM1*) represents the earliest stage of oligodendrocyte development and is expressed in early oligodendrocyte pre-progenitor cells (OPPs). Neuron-glial antigen 2 (NG2; *CSPG4*) is a marker of the next stage of oligodendrocyte development and is expressed in oligodendrocyte progenitor cells (OPCs) and pre-myelinating oligodendrocytes (Pre-OLs). Whilst Galactosylceramidase (*GalC*) is a marker of late oligodendrocytes, and myelin basic protein (*MBP*) a marker of mature, myelinating oligodendrocytes (Shaw *et al*. 2021).

**Figure 7.**
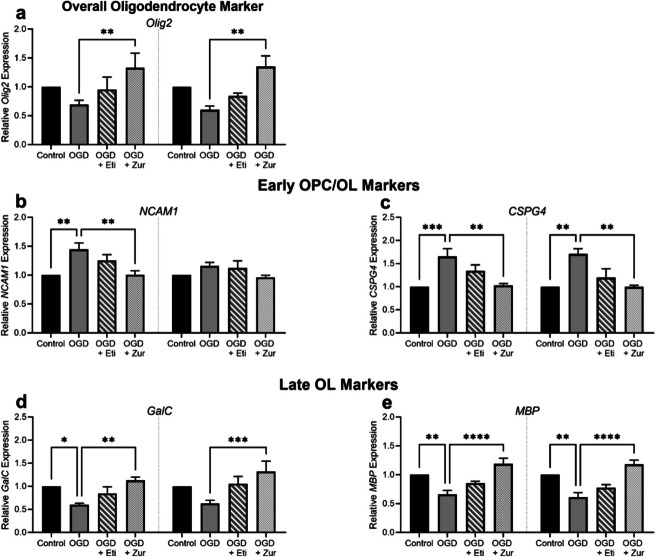
mRNA expression of oligodendrocyte lineage markers following OGD and neurosteroid treatment in oligodendrocytes. Oligodendrocytes are shown as control (*black*; *n* = 5/sex), OGD (*dark grey*; *n* = 5/sex), OGD treated with etifoxine (*striped*; *n* = 5/sex), OGD treated with zuranolone (*light grey* spotted; *n* = 5/sex). Oligodendrocyte mRNA expression includes *Olig2 *(*a*), *NCAM1* (*b*), *CSPG4 *(*c*), *GalC *(*d*) and *MBP *(*e*). Relative mRNA expression is normalized to respective control cells. Data is presented as mean ± SEM with * indicating significance at *p* < 0.05, * indicating significance at *p* < 0.01 and **** indicating < 0.0001.

Oligodendrocyte transcription factor (*Olig2*), a marker of all oligodendrocyte populations, was significantly increased in both male and female zuranolone treated cells compared to insult only (*p* = 0.006, *p* = 0.002, Fig. 7*a*). All subsequent oligodendrocyte lineage markers were normalized to their respective *OLIG2* values prior to normalizing to respective controls to ensure OGD insult related changes were due to the insult and not the overall global change in oligodendrocytes.

Male oligodendrocytes exposed to OGD had a significant increase in *NCAM1* expression compared to control (*p* = 0.002, *p* = 0.002; Fig. 7*b*) which was restored following zuranolone treatment (*p* = 0.002).

A similar response was observed in *CSPG4* expression, where both male and female cultures exposed to OGD saw a significant increase in expression (*p* = 0.0008, *p* = 0.001; Fig. 7*c*). This increase was prevented by zuranolone treatment in male (*p* = 0.001) and female (*p* = 0.007) cells when compared to insult alone.

Following OGD, there was a significant decrease in *GalC* expression in male cultures (*p* = 0.03; Fig. 7*d*) but did not reach significance in females (*p* = 0.12). This decrease was prevented by zuranolone treatment in male (*p* = 0.003) and female (*p* = 0.0004) oligodendrocytes when compared to insult alone.

Similarly, *MBP* expression in both male and female cultures was significantly decreased following OGD exposure (*p* = 0.003, *p* = 0.002; Fig. 7*e*), and again was increased following zuranolone treatment (*p* < 0.0001, *p* < 0.0001).

### Neuroprotective mRNA expression of neurosteroid treatments on oligodendrocytes following OGD

**Figure 8.**
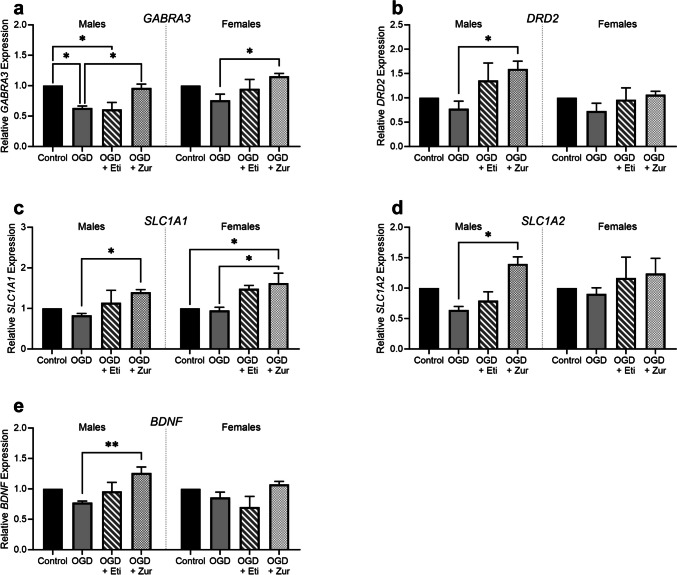
mRNA expression of neuroprotective factors following OGD and neurosteroid treatment in oligodendrocytes. Oligodendrocytes are shown as control (*black*; *n* = 5/sex), OGD (*dark grey*; *n* = 5/sex), OGD treated with etifoxine (*striped*; *n* = 5/sex), OGD treated with zuranolone (*light grey* spotted; *n* = 5/sex). mRNA expression includes GABRA3 (*a*), *DRD2 *(*b*), *SLC1A1 *(*c*), *SLC1A2 *(*d*) and *BDNF *(*e*). Relative mRNA expression is normalized to respective control cells. Data is presented as mean ± SEM with * indicating significance at *p* < 0.05, ** indicating significance at *p* < 0.01, and *** indicating significance at *p* < 0.001.

Neurosteroid treatments had an impact on several other key pathways in the primary oligodendrocytes, which may contribute to the neuroprotective properties of these therapeutics. OGD-exposed male oligodendrocyte cultures had a significant reduction in *GABRA3* expression, which remained reduced with etifoxine treatment (Fig. 8*a*; *p* = 0.01, *p* = 0.01, respectively). In both sexes however, zuranolone treatment significantly increased expression of *GABRA3* compared to insult alone (*p* = 0.04, *p* = 0.01, respectively). mRNA expression of other GABA_A_ receptor subunits was not detected in the oligodendrocyte cultures. *DRD2* expression was found to be significantly increased in male zuranolone treated cultures, compared to insult only, returning expression to control levels (Fig. 8*b*; *p* = 0.01). No changes were observed in females. *SLCA1* was increased in both sexes in zuranolone treated cultures compared to insult only (Fig. 8*c*; *p* = 0.03, *p* = 0.02). *SLC1A2* expression was found to be significantly increased in male zuranolone treated cultures, compared to insult only (Fig. 8*d*; *p* = 0.01). Similarly, *BDNF* expression was also increased in male zuranolone treated cultures, compared to insult only (Fig. 8*e*; *p* = 0.002).

## Discussion

In this study, we examined the effects of neurosteroid treatments against oxygen–glucose deprivation in both primary neurons and oligodendrocytes as a model of preterm birth associated hypoxia–ischemia. We investigated the effects of etifoxine; a TSPO agonist that also binds directly to the GABA_A_ receptor, as well as zuranolone; a synthetic analogue of allopregnanolone. Overall, the present findings suggest that zuranolone had actions through suppression of excitability and etifoxine by triggering several supportive actions. The findings showed that OGD induced a significant increase in cytotoxicity in primary neurons and oligodendrocytes as is seen of hypoxia in the preterm newborn, supporting the relevance of the OGD injury model. The reversal of many of the OGD-induced changes by zuranolone and etifoxine further suggests these treatments may be valuable therapies. Importantly, the observation of primary neurons showing reductions to key neuronal markers, which were either restored to control with etifoxine or significantly increased with zuranolone, indicates novel pathways may contribute to the protective actions of these treatments. Both drug treatments appeared to target specific GABA_A_ receptors and glutamate receptors, as well as transporters and synthesis enzymes, suggesting that the mechanism of and subsequent prevention of damage occurred through a specific receptor driven manner. Oligodendrocyte cultures exposed to OGD appeared to have arrested mRNA expression of key markers of the oligodendrocyte lineage, which were restored with both neurosteroid treatments. Additionally, key neuroprotective markers were also enhanced in the oligodendrocyte cultures, matching the changes observed in the neuronal cultures.

The effects of OGD in neuronal cultures were further investigated through expression of key neuronal markers. The finding that *RBFOX3*, a marker of overall neurons, was reduced with OGD in both males and females’ mimics what is seen clinically, as preterm birth and hypoxic-ischemic events are associated with reductions to grey matter (Lear *et al*. 2023). Additionally, *PVALB*, a key GABAergic interneuron marker, appeared to be the most affected by OGD compared with the other GABAergic interneuron markers examined. Parvalbumin is key to regulating the excitatory-inhibitory balance in the brain through controlled timing of pyramidal cellular output, particularly within the frontal cortex (Isaacson and Scanziani 2011; Miyamae *et al*. 2017). Interestingly males appeared particularly vulnerable to damages to somatostatin (*SST*), another key interneuron marker. Males were again observed as being disproportionately impacted by changes in mRNA expression of *BDNF*, a neurotrophic factor critical for neuronal cell survival. This suggests that even at the cellular level, there may be protective mechanisms employed in female cultures that reduce the impact of OGD. Previous studies have also observed male neuronal rodent cultures to be more sensitive to damages than females and suggest that the sexually dimorphic protein soluble epoxide hydrolase may be contributing to these differences (Fairbanks *et al*. 2012). Zuranolone provided a robust increase in all neuronal markers examined, while etifoxine restored most parameters back to control, suggesting potential differences in the mechanisms underpinning these neurosteroid treatments. This may involve OGD potentially targeting TPSO pathways, and therefore limiting the efficacy of this agent.

We speculate that underlining mechanisms of neuroprotection by neurosteroid therapies such as etifoxine and zuranolone involve the modulation of the GABAergic and glutamatergic pathways following receptor stimulation. In the male neuronal culture population, we found that *GAD67* (rate limiting enzyme in the production of GABA from glutamate) expression was significantly increased following both etifoxine and zuranolone treatment, under OGD conditions, compared to insult only cells. It is well established that OGD has a high propensity to increase glutamate release and result in imbalanced excitation (Martin *et al*. 1994; Fujimoto *et al*. 2004; Helleringer *et al*. 2017). Increased mRNA expression of *GAD67* following treatment could potentially help buffer frontal cortex neurons against unbalanced excitation by allowing for a greater production of GABA to antagonise the increased glutamate release. In females we observed a similar effect of treatment following OGD, however only zuranolone treatment increased *GAD67* significantly in this population. Meanwhile, in both sexes we saw a significant increase in the expression of *SLC1A2* (reuptake of glutamate from the synaptic cleft and extracellular space) expression following zuranolone treatment under OGD-induced conditions. Increased expression of *SLC1A2* suggests that there is an increased rate of clearance of glutamate from the synapse and extracellular space which would likely buffer these neurons from imbalanced excitation. Following zuranolone treatment these increases in *GAD67* and *SLC1A2* may act synergistically, by providing increased substrate for the *GAD67* enzyme to convert into GABA, thereby decreasing synaptic glutamate and increasing the availability of the inhibitory neurotransmitter, GABA. These observations suggest zuranolone enhances GABA availability as well as raising GABAergic stimulation when buffering against OGD-induced excitotoxicity. These additional actions may underpin the greater efficacy in improving survival following OGD.

Excessive glutamatergic action is a classic hallmark of excitotoxic damage, which is frequently observed in conditions of preterm hypoxia–ischemia (Johnston 2001). *GRIA1* (GLUA1 AMPA receptor subunit) expression was found in male neurons to increase following OGD, compared to control neurons and zuranolone treated, OGD neurons. Previous evidence suggests that increased excitatory activity and long-term potentiation (LTP) can raise the expression of GLUA1 containing AMPA receptors at the synapse (Hayashi *et al*. 2000; Shi 2001; Qu *et al*. 2021). OGD has a high propensity to increase neuronal activity through imbalanced excitation and this likely increases the synaptic concentrations of GLUA1 subunit containing AMPA receptors. Alternatively, we observed no changes in the mRNA expression of *GRIA2* (GLUA2 AMPA receptor subunit) for either sex. Interestingly, GLUA2 forms calcium impermeable AMPA receptors which are a neuroprotective variant of the receptor (Wright and Vissel 2012). Therefore, increased *GRIA1* in the context of static *GRIA2* expression suggests that OGD increases the ratio of calcium permeable AMPA receptor expression, which is prevented following zuranolone treatment. Zuranolones capacity to prevent increases in *GRIA2* following OGD suggests that it protects against AMPA mediated excitotoxicity, thereby contributing to neuronal survival by preventing the excessive stimulation of glutamate receptors. Similarly, it appears that zuranolone protects against NMDA receptor mediated excitotoxicity as the OGD-induced increases of *GRIN1* (GluN1 NMDA receptor subunit) and *GRIN2A* (GluN2A NMDA receptor subunit) were prevented by zuranolone treatment. Increases in NMDA receptors expressing the GluN2A subunit results in a higher propensity for channel opening and faster recovery from desensitisation (Vicini *et al*. 1998; Chen *et al*. 1999; Franchini *et al*. 2020). Whilst extrasynaptic glutamate receptors are typically implicated in excitotoxicity, GluN2A containing receptors are typically expressed in the synapse (Petralia 2012; Franchini *et al*. 2020) and previous studies show that synaptic NMDA receptors are associated with benefits to neuronal survival (Luo *et al*. 2011). Therefore, increased *GRIN2A* following OGD may indicate an increased requirement for synaptic NMDA receptors which confer a pro-survival effect on the neuron. Zuranolone, which increases GABAergic tone through binding to GABA_A_ receptors, prevents the increase of *GRIN2A* expression following OGD, likely through decreasing excitation, thereby protecting against excitotoxicity without the need for increased synaptic NMDA receptor signalling.

As etifoxine and zuranolone have been previously shown to modulate GABAergic activity, and specifically GABA_A_ receptor expression (Hamon *et al*. 2003; Althaus *et al*. 2020), in the current study we examined mRNA expression of key GABA_A_ receptor subunits in the neuronal cultures. Etifoxine increased *GABRA1* expression, which is a key subunit involved in the sleep–wake cycle (Lie *et al*. 2021). GABA_A_R4 and GABA_A_Rδ subunits are both extra-synaptic GABA_A_ receptor subunits, which mediate tonic inhibition and are highly sensitive to neurosteroids (Wang 2011). Consistent with previous work, etifoxine and zuranolone appeared to enhance *GABRA4* expression, whereas *GABRAD* was primarily impacted by zuranolone. As mentioned, extrasynaptic GABA_A_ receptor enhancement has been proposed to dampen glutamate release and subsequent excitotoxicity related damages (44). Thus, the current findings suggest that effects on subunit expression are a key part of the mechanism behind the neuroprotection observed in this study. Interestingly, both neurosteroid treatments appeared to also interact with the expression of the two key dopamine receptors. Both etifoxine and zuranolone increased *DRD1* expression following OGD in both sexes, indicating that GABAergic stimulation may also prevent reductions in dopamine mRNA expression. Zuranolone is currently in clinical trials for tremors associated with Parkinson’s disease, as neurosteroid treatments may represent a novel adjunctive therapy for this condition (Bullock *et al*. 2021). Interestingly, we observed sex differences which, whilst not significant, trended towards decreased *DRD2* expression, which was restored with concurrent etifoxine and zuranolone treatment. Sex differences have been found previously in *DRD2* levels, particularly in the mesocortical region, which also may explain increased predisposition of males towards dopamine related disorders such as attention deficit hyperactivity disorder (ADHD) (Andersen and Teicher 2000; Kaasinen *et al*. 2001).

Lastly, OGD appeared to arrest the maturation and differentiation of the cells in oligodendrocyte cultures. This is evidenced by increased expression of the earlier oligodendrocyte lineage stages (*NCAM1* and *CSPG4)* following OGD, indicating that there is a higher proportion of these cells that did not continue to mature through the lineage. Furthering this is the reduction of the later oligodendrocyte and myelin markers (*GalC* and *MBP)* following OGD. These findings are in line with previous literature that suggests alterations to white matter following preterm birth occur due to arrested maturation of the oligodendrocyte lineage (Buser *et al*. 2012), again strengthening the value of this model. Importantly, concurrent treatment with Zuranolone improved the maturation of these cell markers, which is consistent with previous studies suggesting a trophic role for allopregnanolone (48) which is likely mediated by GABA_A_ receptor stimulation. This enhanced maturation of oligodendrocyte cells is particularly important in the case of preterm birth brain injury, with the well-established increased risk of diffuse white matter injury in preterm neonates and its known link to neurobehavioral disorders (Reyes-Haro *et al*. 2021). Neurosteroid treatments also appear to modulate key targets in oligodendrocyte cultures in a similar manner to neuronal cells. *GABRA3* was reduced in male oligodendrocytes but not female, indicating further sex differences in protective processes, with zuranolone again normalising these levels. Increased *DRD2* expression with zuranolone treatment may also be a key mechanism involved in the maturation of oligodendrocytes observed here, as these receptors have been proposed to play pivotal roles in maturing and differentiating oligodendrocytes. Previous studies investigating primary oligodendrocyte cultures showed that dopamine D2 and D3 agonists were neuroprotective against glutamatergic toxicity (Rosin *et al*. 2005). This may be occurring through to the observed increase in mRNA expression of glutamatergic transporters *SLCA1* and *SLCA11* in this study, which both reduce extracellular glutamate levels. Finally, similar to the neuronal cultures, *BDNF* expression was also increased in oligodendrocytes with zuranolone treatment, which is proposed to play roles in the maturation of these cells (VonDran *et al*. 2011). It is worth noting that preterm birth has previously been shown to disrupt other glial populations, including the activation of microglia and upregulation of pro-inflammatory astrocytes (Baburamani *et al*. 2014; Mallard *et al*. 2014). Neurosteroid therapies have been shown to exert anti-inflammatory effects both preclinically and clinically, suggesting that some of the neuroprotective effects may be attributed to the modulation of other glial cells (Murugan *et al*. 2019; Balan *et al*. 2023).

In conclusion, the current observations highlight the potential of neurosteroid therapies for the treatment and prevention of preterm brain injuries. The studies suggest key mechanisms underlying these neuroprotective effects involving GABAergic and glutamatergic pathways. These changes in receptor, enzyme and neurotrophic factor mRNA expression provide a solid base for further work in this area, particularly to ensure these changes are translated into the protein level. Despite this limitation, we observed marked changes in cytotoxicity in this model and are confident that these neurosteroid treatments have a marked beneficial impact on neuronal survival and oligodendrocyte maturation. Future studies staining for specific neuronal and oligodendrocyte markers might be useful to demonstrate these mRNA changes do translate into protein. Additionally, the assessment of these neuroprotective therapies on other glial cells, such as microglia and astrocytes, would provide further insight into the mechanisms that are occurring. Further investigation is also warranted in moving these therapies to in vivo work, to examine if these mechanistic changes lead to improved long term neurobehavioural outcomes. Given the persistent high rate of preterm births and current limited treatment options, neurosteroid treatments may be a potential therapy in preventing the long-term impairments after preterm birth due to the combined effects of deficits and excitotoxicity in the developing brain.
